# How does image quality affect radiologists’ perceived ability for image interpretation and lesion detection in digital mammography?

**DOI:** 10.1007/s00330-020-07679-8

**Published:** 2021-01-21

**Authors:** Joana Boita, Ruben E. van Engen, Alistair Mackenzie, Anders Tingberg, Hilde Bosmans, Anetta Bolejko, Sophia Zackrisson, Matthew G. Wallis, Debra M. Ikeda, Chantal Van Ongeval, Ruud Pijnappel, Mireille Broeders, Ioannis Sechopoulos, for the VISUAL group, F. Jansen, L. Duijm, H. de Bruin, I. Andersson, C. Behmer, K. Taylor, F. Kilburn-Toppin, M. van Goethem, R. Prevos, N. Salem, S. Pal

**Affiliations:** 1 Department of Medical Imaging, Radboud University Medical Center, Geert Grooteplein 10, 6525 GA Nijmegen, The Netherlands; 2 Dutch Expert Centre for Screening (LRCB), Wijchenseweg 101, 6538 SW Nijmegen, The Netherlands; 3 National Coordinating Centre for the Physics of Mammography, Royal Surrey NHS Foundation Trust, Guildford, GU2 7XX UK; 4 Department of Medical Radiation Physics, Translational Medicine Malmö, Lund University, Skåne University Hospital, Carl Bertil Laurells gata 9, SE-20502 Malmö, Sweden; 5 Department of Imaging and Pathology, Radiology, KUL, Herestraat 49, B-3000 Leuven, Belgium; 6 Department of Radiology, Radiology, UZ Gasthuisberg, Herestraat 49, B-3000 Leuven, Belgium; 7 Department of Medical Imaging and Physiology, Translational Medicine Malmö, Lund University, Skåne University Hospital, Carl Bertil Laurells gata 9, SE-20502 Malmö, Sweden; 8 Cambridge Breast Unit, Cambridge University Hospitals NHS Foundation Trust, Cambridge & NIHR Cambridge Biomedical Research Centre, Cambridge, CB2 0QQ UK; 9 Department of Radiology, Stanford University School of Medicine, 875 Blake Wilbur Dr, Stanford, CA 94305 USA; 10 Department of Radiology, University Medical Center Utrecht, Utrecht University, PO Box 85500, 3508 GA Utrecht, The Netherlands; 11 Department for Health Evidence, Radboud University Medical Center, Geert Grooteplein 10, 6525 GA Nijmegen, The Netherlands

**Keywords:** Breast cancer, Digital mammography, Perception, Quality control

## Abstract

**Objectives:**

To study how radiologists’ perceived ability to interpret digital mammography (DM) images is affected by decreases in image quality.

**Methods:**

One view from 45 DM cases (including 30 cancers) was degraded to six levels each of two acquisition-related issues (lower spatial resolution and increased quantum noise) and three post-processing-related issues (lower and higher contrast and increased correlated noise) seen during clinical evaluation of DM systems. The images were shown to fifteen breast screening radiologists from five countries. Aware of lesion location, the radiologists selected the most-degraded mammogram (indexed from 1 (reference) to 7 (most degraded)) they still felt was acceptable for interpretation. The median selected index, per degradation type, was calculated separately for calcification and soft tissue (including normal) cases. Using the two-sided, non-parametric Mann-Whitney test, the median indices for each case and degradation type were compared.

**Results:**

Radiologists were not tolerant to increases (medians: 1.5 (calcifications) and 2 (soft tissue)) or decreases (median: 2, for both types) in contrast, but were more tolerant to correlated noise (median: 3, for both types). Increases in quantum noise were tolerated more for calcifications than for soft tissue cases (medians: 3 vs. 4, *p* = 0.02). Spatial resolution losses were considered less acceptable for calcification detection than for soft tissue cases (medians: 3.5 vs. 5, *p* = 0.001).

**Conclusions:**

Perceived ability of radiologists for image interpretation in DM was affected not only by image acquisition-related issues but also by image post-processing issues, and some of those issues affected calcification cases more than soft tissue cases.

**Key Points:**

*• Lower spatial resolution and increased quantum noise affected the radiologists’ perceived ability to interpret calcification cases more than soft tissue lesion or normal cases.*

*• Post-acquisition image processing-related effects, not only image acquisition-related effects, also impact the perceived ability of radiologists to interpret images and detect lesions.*

*• In addition to current practices, post-acquisition image processing-related effects need to also be considered during the testing and evaluation of digital mammography systems.*

**Supplementary Information:**

The online version contains supplementary material available at 10.1007/s00330-020-07679-8.

## Introduction

Digital mammography (DM) is the predominant imaging technique used for breast cancer screening. However, image interpretation and detection of breast lesions will be affected by the quality of the acquired mammogram [1–8]. Image quality refers to the collection of image parameters, such as signal-to-noise ratio, spatial resolution, and contrast, whose levels need to be high enough to have a mammogram that allows the interpreting radiologist to distinguish pathological structures from the background, i.e., resulting in a mammogram that fulfils its diagnostic purpose. Therefore, evaluation of image quality is an important procedure to identify and understand the issues that may affect image interpretation. For this reason, guidelines were developed to assess, optimise, and accept digital mammography systems, with the aim of maximising breast screening performance [9]. An example of evaluation procedures of DM systems is type testing, where both the technical and clinical performance of a new DM system are assessed before their clinical introduction [9].

There are various sources of potential image quality issues that can affect mammographic image interpretation and that are taken into account in these guidelines. Considering the very different characteristics between the two major types of findings suggesting cancer in mammography (soft tissue lesions and calcifications), the imaging issues that may hamper their detection and interpretation, in terms of size, density, margin, distribution, and shape, could differ. For soft tissue lesions, for example, shape, margin characteristics, and overall density are important discriminators between benign and malignant lesions. For calcifications, number, morphology, and distribution are important features to consider during their interpretation [10, 11]. Therefore, soft tissue lesion visibility can be affected by the characteristics in the depiction of the breast anatomical structure, such as contrast, while calcification detectability can be affected by the presence of noise, such as random quantum noise, which generally limits the visibility of small structures [12–15]. However, a relatively higher noise level in the image, due to, e.g., a reduced radiation dose, can also affect the discrimination of benign and malignant soft tissue lesions [13–15]. Given the relationship between these image quality features and the image acquisition process, image quality issues such as relatively higher noise levels can be a consequence of suboptimal selection of acquisition settings, suboptimal settings of the automatic exposure control, or of problems with the x-ray source of the mammography system.

Image blurring is another issue that can lead to a reduction in lesion detection performance, although lesions of different types may be impacted to a different degree. Blurring can be a consequence of, for instance, breast motion, the resolution properties of the detector, or the imaging geometry, including the effective size of the x-ray focal spot [16, 17]. Some studies show that lower system resolution affects calcification visibility more than soft tissue lesion visibility, mainly because calcifications are fine and small structures, making it more difficult to distinguish their morphology, distribution, and number, in images with higher blur [17, 18]. Meanwhile, when comparing the impact of higher noise levels and lower spatial resolution on lesion detectability, the former seems to affect detection accuracy more than the latter, for both types of lesions [18].

Ideally, the application of any image processing algorithm should provide the maximum possible amount of useful information present in the mammogram to the radiologists. In this way, the visual appearance of the mammogram is modified, allowing for an improved visibility of the breast structures and the lesions for a human reader, by optimising the displayed brightness and contrast throughout the image. However, it has been shown that this depends on the image processing methods used, which may impact soft tissue lesion and calcification detection differently [6–8]. There are innumerable image processing methods implemented by manufacturers, which change the visual appearance of mammograms differently [5, 6]. However, these implementations are proprietary, with little to no information about these processes made public. Therefore, it is not straightforward to predict how image processing actually changes the visual appearance of mammograms and how it impacts clinical performance. Inadequate image processing algorithms may result in the contrast being increased, or decreased too much, negatively affecting, for instance, the depiction of the differences in density of breast structures and breast lesions. Furthermore, some processing algorithms may increase the spatial correlation of the pixels in the image, creating changes in the texture of the breast that could resemble calcifications.

Consequently, it is essential that comprehensive image quality evaluation considers both image acquisition and processing to ensure the optimal performance of systems, in such a way that neither the detection nor interpretation tasks by the radiologists are negatively affected. This means that the full imaging chain needs to be tested and controlled in order to guarantee a sufficient quality of mammograms. However, to ensure the clinical relevance of image quality evaluation, it is important to understand how image quality affects lesion detection and interpretation from the radiologists’ point of view.

Therefore, the goal of this study was to investigate how different image quality issues affect the perceived ability of radiologists for image interpretation and lesion detection in digital mammography.

## Materials and methods

In brief, previously acquired mammograms were degraded in five different ways separately, to six different levels of degradation. While reviewing the reference image and the images with the six levels of degradation, radiologists, knowing the lesion location, were asked to select the mammogram of the lowest quality they still felt was just acceptable for interpretation.

### Digital mammography cases

A total of 45 mammographic cases, with breast density varying from fatty to dense, and with and without lesions, were selected and retrieved, under license, from the OMI-DB anonymised database of mammograms, which is part of the OPTIMAM project [19]. Given the use of this existing anonymised database, no ethics approval was needed for this study. This set contained a total of 44 lesions: 30 cancers and 14 benign findings, in addition to 5 normal cases, all with pathological (in case of the lesion-containing cases) or follow-up confirmation. Four out of the 45 cases had two lesions present, one in each breast. The cases with lesions, as well as the respective lesion locations, were reviewed and annotated by an expert radiologist. The positive cases contained different types of lesions, as described in Table 1, in order to include most types of lesions found at mammography. All images were acquired using Lorad Selenia (Hologic, Inc.) mammography systems. The original, reference, mammograms were all determined to be adequate for interpretation, but not necessarily perfect, as is the case with the majority of acquired mammographic images. Each case consisted of four views. From these, the view in which the lesion features (when present) were most visible was chosen to be degraded. In case of lesion absence, the view to be degraded was chosen at random.

**Table 1:** Breakdown of the 44 lesions included in the image set, by type

	Benign (*n* = 14)	Malignant (*n* = 30)
Ill-defined mass	3	4
Well-defined mass	4	1
Spiculated mass	-	8
Asymmetry	-	5
Architectural distortion	-	6
Calcification	7	6

### Image degradation procedure

**Fig. 1:**
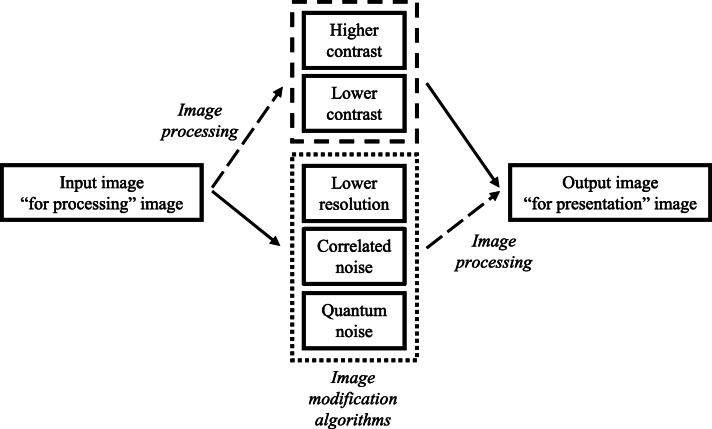
Pipeline of the image quality modification. In the case of contrast modifications, the “for presentation” image was degraded. In the case of reducing the resolution or modifying the noise, the “for processing” image was modified first and then processed. In all cases, the final output was “for presentation” images

Previously developed algorithms [20–22] and algorithms specifically developed for this study were used to decrease the quality of mammograms by introducing different image quality issues in the images. The types and magnitude of image quality issues were selected based on previously observed issues in digital mammography systems submitted for type testing. The introduced image quality issues lower spatial resolution, increased or decreased contrast, increased pixel correlation (denoted by correlated noise), and increased random quantum noise in the image (lower dose). Lower spatial resolution and increased quantum noise represented problems caused by issues in the acquisition performance of the mammography system, while correlated noise and contrast issues represented problems caused by the post-acquisition image processing. A pipeline of the image quality modification is shown in Fig. 1.

For each of the five simulated image quality issues, six images with increasing levels of degradation were generated from the reference image. The levels were selected so that the structures and lesions in the image were not exaggeratedly degraded, according to what is clinically relevant and realistic.

As described in Fig. 1, anonymised “for processing” images, after (lower resolution and quantum and correlated noise) or before (higher and lower contrast) image quality modification, were processed and converted into “for presentation” images using an off-line version of the image processing applied by the reference imaging system. Details on the image degradation processes can be found in the online supplement.

### Radiologist review

Reviews of the selected mammographic cases were conducted by fifteen breast radiologists, from five different countries (UK, USA, Sweden, Belgium, and the Netherlands), that do both screening and clinical work. The radiologists had a median of 20 years (range: 5–46 years) of experience in reading mammograms. Cases were viewed either on calibrated 5- or 12-megapixel liquid crystal high-luminance mammography displays (Coronis 5MP Mammo and Coronis Uniti, Barco). The images were displayed according to the DICOM standard for presentations using ViewDEX [23], software specifically developed for observer studies. The software allowed the radiologists to move forward and backward between cases, and to zoom, pan, and scroll over the images in each case.

When first presented with a new case, the location of the lesion on the displayed image was indicated to the radiologists, and the seven mammograms were shown in order of decreasing image quality. The images were labelled with a numerical index ranging from 1 (reference) to 7 (most degraded), corresponding to the degradation levels. The first image of each set of images was the reference image, i.e., the original image without any change in quality. The radiologists were asked to select the mammogram of the lowest quality that they still felt was acceptable for interpretation. At the selected degradation level, the image should still allow the radiologist to interpret the image and to look for other lesions in the surrounding breast tissue with confidence. In case of lesion presence, the image should also allow the radiologists to detect and interpret the lesion.

### Analysis

The median (M) and distribution of the median of the radiologist index of the threshold images selected by each radiologist were calculated for each type of degradation according to lesion type, including the normal cases. All soft tissue lesion cases (masses, asymmetries, and architectural distortions) were combined in one group and all calcification cases were combined in another group. The normal cases without lesions were added to the first group, since the general features that the radiologists considered while assessing the soft tissue lesions and the surroundings were approximately the same: linear structures, density, and margins, being then denoted as soft tissue cases. The two-sided, non-parametric Mann-Whitney test was conducted using R (version 3.6.3, R Foundation for Statistical Computing) to test for differences in the radiologists’ perceived ability to interpret calcification and soft tissue cases for each type of degradation by comparing the medians of each radiologist indices. *p* < 0.05 was used to indicate statistical significance for this analysis.

## Results

**Fig. 2:**
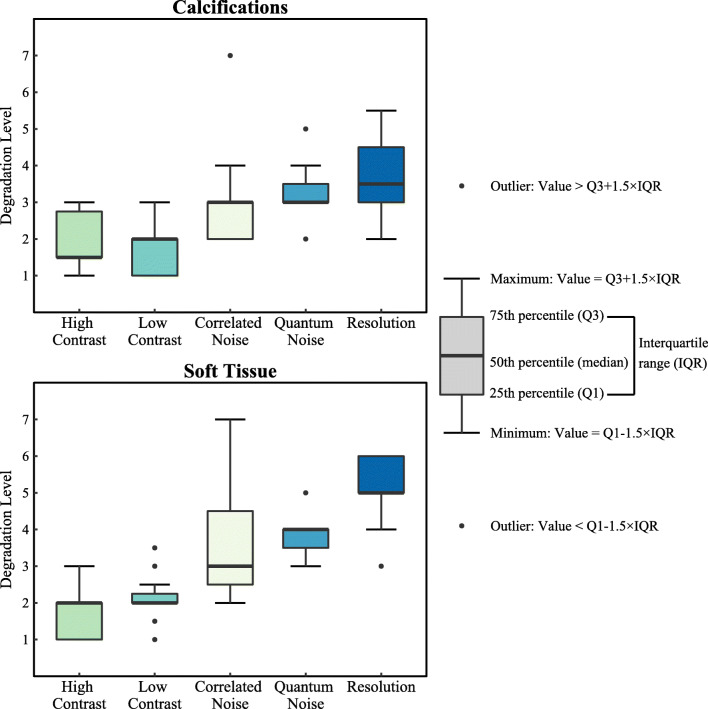
Distribution of the median of the radiologist index (1–7) for the lowest acceptable level of image quality by degradation type, corresponding index 1 to reference image, for (upper) calcifications and (lower) soft tissue cases. Boxplot explanation (right)

Figure 2 shows the distribution of the median index of the threshold images selected by each radiologist for calcification cases and soft tissue cases. The index variability results for each case type can be found in Table 1S in the online supplement. The radiologists perceived to be more sensitive to lower spatial resolution and increased quantum noise in calcification-containing images than when evaluating images containing soft tissue lesions and normal cases (*p* = 0.001 and *p* = 0.02, respectively).

**Fig. 3:**
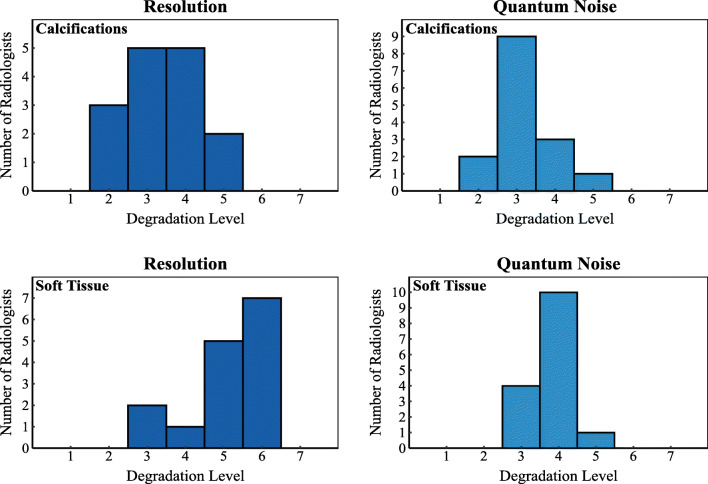
Histogram corresponding to the levels of degradation (1–7, corresponding index 1 to reference image) that radiologists selected as still acceptable in images with lower resolution and quantum noise for (top row) calcification cases; and (bottom row) soft tissue cases

As can be seen for the images with higher contrast, for both calcification and soft tissue cases, most radiologists selected as median threshold images the ones degraded with the first levels of degradation (*M* = 1.5 and *M* = 2, respectively). The same was observed for images with lower contrast (*M* = 2, for both types of cases). Degradation by addition of correlated noise was tolerated to a higher degree (*M* = 3, for both types of cases). When it comes to images with increasing quantum noise, the boxplot shows that most radiologists selected the images with a 30% (*M* = 3) and 45% (*M* = 4) of dose reduction, for calcification and soft tissue cases, respectively, as the median threshold images. Considering the images with lower spatial resolution, for both types of cases, most radiologists selected the median threshold images to be *M* = 3.5 and *M* = 5, corresponding to the spatial resolution equivalent to that of specific indirect digital and computed radiography mammography systems (see system specifications in the online supplement). This distribution, for both types of cases, is shown in more detail in the histograms of the radiologist index for images with lower resolution and quantum noise in Fig. 3.

**Fig. 4:**
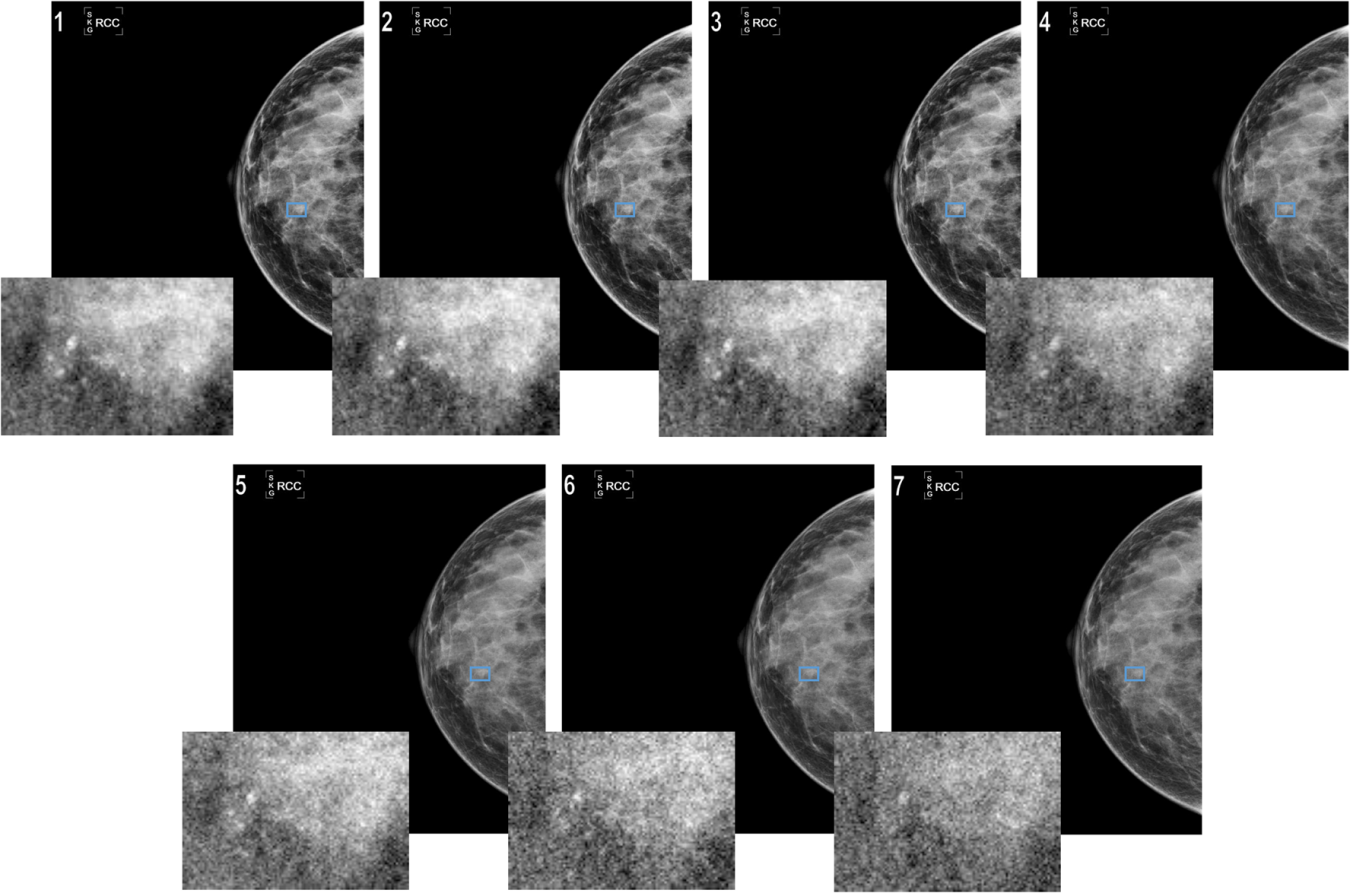
Effects of increasing quantum noise in the image corresponding to a case with calcifications

Figure 4 shows an example of the effects of increasing quantum noise in the image corresponding to a case with calcifications.

## Discussion

Lower image quality substantially affects image interpretation and the detection of breast cancer in digital mammography [1–8]. Therefore, it is important to identify and understand the potential image quality-related issues that may impact the visibility of lesions, in order to, for instance, design more effective testing procedures, such as type testing. Additionally, it can also be important to optimise DM system performance and guarantee adequate image quality, and to set better and more evidence-based limiting values during evaluation of DM systems. This will ensure that the issues that actually affect clinical performance are the ones evaluated with relevant limiting values.

In this study, five image quality issues that were expected to lower the perceived ability of radiologists for image interpretation and lesion detection in digital mammography were evaluated. The effect of lower spatial resolution and increased quantum noise on radiologists’ perceived ability was worse in calcification cases than in soft tissue cases (including soft tissue lesion and normal cases) (Fig. 3), as also observed in previous studies [14, 17, 18]. This probably occurs due to the fact that calcifications are very small and can easily disappear when the images are affected by these two low-image quality effects. Furthermore, the different types of degradation affected the calcification cases in a more consistent manner than they did the soft tissue cases. Therefore, the conclusion that calcifications and soft tissue are affected differently by changes in image quality [8, 24] was once again confirmed and supported by this study. The fact that soft tissue cases were affected differently than the calcification-containing cases is a key result, which calls into question the assumption that “the visibility of normal anatomy is strongly correlated to the detectability of pathological structures” [25]. This assumption, which has been used for previous studies to evaluate image quality and is a common assumption in the design of clinical evaluations in type testing, is not necessarily applicable to calcification depiction. Therefore, system testing needs to evaluate not only the depiction of normal anatomy but also the impact of system behaviour on pathological structures.

Thresholds of acceptability of the mammograms affected by each degradation type were identified in this study. In terms of spatial resolution, the capabilities of computed radiography-based mammography systems were considered less acceptable by most of the radiologists in the depiction of calcifications. Meanwhile, soft tissue lesions were not affected substantially by decreases in spatial resolution, because they are larger structures. These findings agree with previous studies that conclude that CR-based mammography systems perform worse than DM systems for calcification detection [12, 16, 24, 26]. The increase in quantum noise resulting from simulated dose reduction was not tolerated past the selected threshold due to the introduction of a grainy effect in the breast tissue texture, as commented by the radiologists. In this study, the radiologists were more confident with dose reductions than those found by Warren et al [12]. However, in their work, both image set and study design were different from the ones used in this work; it consisted of a detection task study, without knowing in advance the lesion location, which also contributed to the verified differences between the two studies. For the images with lower and higher contrast, the level of degradation that the radiologists still felt acceptable was low. This means that the reference images could not be decreased much in quality before they were deemed not acceptable. In cases of increasing the image contrast, the reference images were already of very high-contrast, with the soft tissue locally enhanced. Therefore, this result could have been different with images from a different system manufacturer. Also, the way how contrast was adapted, by increasing it globally in the image, was different from how it was adapted by the manufacturer, who enhances it locally. This might lead to a narrow range of acceptable degradation levels. In the cases with reduced contrast, the radiologists lost confidence already with the first degradation levels, because the whole image was affected, yielding a “flat” image. Therefore, some structures and respective features were not well visualised. The increase in noise correlation, which is a pitfall of image processing, was also not highly tolerated, as commented on by the radiologists, because of the introduction of simulated signals that could resemble calcifications. This explains why some highly degraded images were still acceptable in the soft tissue cases.

It was shown that issues such as the change of contrast and correlated noise, which are related more to post-acquisition image processing effects than image acquisition, have a high impact on the radiologists’ perceived ability to interpret mammograms. This means that evaluation procedures such as type testing should take into account the post-acquisition image processing effects during the clinical evaluation phase. The European guidelines for quality assurance in breast cancer screening and diagnosis [9] include an evaluation procedure in which radiologists are asked to score a set of criteria about visibility of certain structures in processed images. However, this procedure is not a direct assessment of the image processing algorithms; it is only an approximate attempt to evaluate the impact of image processing effects on image quality. Therefore, the thresholds of acceptability of images for each degradation type identified in this study cannot be considered limiting values for mammography system testing, but they can provide relevant information that can be used in future studies for image quality evaluation and that will help in the evaluation of image processing algorithms.

This study also has some limitations. First, the degradation of image quality was simulated, not obtained with actual problematic mammography systems. Also, when simulating different levels of lower resolution, the different systems were not fully simulated; only one parameter, the sharpness, was considered. However, these simulation methods have been validated in previous phantom-based studies [20–22, 27]. Also, as in virtual clinical trials, these methods allowed for simulating of different image quality degradation types and levels applied to the same case, which would be impossible to obtain in the clinical setting. By using simulations, these images were obtained without the need for repeated radiation exposure of human subjects and allowing for selection of cases with specific characteristics, which can be a difficult task otherwise. Second, the magnitude of the degradation might be different across the different types of degradation; i.e., in some cases, the image quality range covered by one degradation type was larger than for other types. For instance, the spatial resolution levels were a representation of different systems found in the clinical setting. Therefore, the difference between the levels may not have been as substantial as in other types of degradation. This made impossible the comparison across degradation types. However, the levels for each degradation type were selected according to what is clinically relevant and realistic, and the results across lesion type, within a degradation type, could be compared. Finally, the number of mammographic cases included is low. However, we did include a substantial number of radiologists, which ameliorated this limitation. This can be seen by the fact that the distribution of the radiologist indices across cases for each type of degradation did not vary considerably, as shown in Table 1S in the online supplement.

In conclusion, this study showed how different issues in image quality affect the perceived ability of radiologists for image interpretation and lesion detection in digital mammography, demonstrating that acceptability of the mammograms was determined not only by image acquisition-related issues but also by image post-processing issues, which are currently not typically evaluated during system testing.

## Supplementary information

ESM 1 (DOCX 180 kb)

## Abbreviations

CR: Computed radiography
DM: Digital mammography
M: Median

## References

[1] Taplin SH, Rutter CM, Finder C, Mandelson MT, Houn F, White E (2002) Screening mammography: clinical image quality and the risk of interval breast cancer. AJR Am J Roentgenol 178:797–803 10.2214/ajr.178.4.1780797 11906848

[2] Feig SA (2002). Image quality of screening mammography: effect on clinical outcome. AJR Am J Roentgenol.

[3] Eklund GW, Cardenosa G, Parsons W (1994). Assessing adequacy of mammographic image quality. Radiology.

[4] Van Ongeval C, Bosmans H, Van Steen A (2005). Current challenges of full field digital mammography. Radiat Prot Dosimetry.

[5] Pisano ED, Cole EB, Hemminger BM (2000). Image processing algorithms for digital mammography: a pictorial essay. Radiographics.

[6] Zanca F, Jacobs J, Van Ongeval C (2009). Evaluation of clinical image processing algorithms used in digital mammography. Med Phys.

[7] Cole EB, Pisano ED, Zeng D (2005). The effects of gray scale image processing on digital mammography interpretation performance. Acad Radiol.

[8] Warren LM, Given-Wilson RM, Wallis MG (2014). The effect of image processing on the detection of cancers in digital mammography. AJR Am J Roentgenol.

[9] van Engen R, Bosmans H, Heid P, Perry N, Broeders M, de Wolf C (2013). Digital mammography update. European protocol for the quality control of the physical and technical aspects of mammography screening. S1, Part 2: European type testing. European guidelines for quality assurance in breast cancer screening and diagnosis. Fourth edition, Supplements.

[10] Burgess AE (2005) Effect of detector element size on signal detectability in digital mammography. In: Proceedings of SPIE. pp 232–242

[11] Kopans DB (2007) Breast imaging. Lippincott Williams & Wilkins

[12] Warren LM, Mackenzie A, Cooke J (2012). Effect of image quality on calcification detection in digital mammography. Med Phys.

[13] Timberg P, Baath M, Andersson I, Mattsson S, Tingberg A, Ruschin M (2012) Visibility of microcalcification clusters and masses in breast tomosynthesis image volumes and digital mammography: a 4AFC human observer study. Med Phys 39:2431–2437 10.1118/1.3694105 22559613

[14] Ruschin M, Timberg P, Båth M (2007). Dose dependence of mass and microcalcification detection in digital mammography: free response human observer studies. Med Phys.

[15] Samei E, Saunders RS, Baker JA, Delong DM (2007). Digital mammography: effects of reduced radiation dose on diagnostic performance. Radiology.

[16] Yaffe MJ, Bloomquist AK, Hunter DM (2013). Comparative performance of modern digital mammography systems in a large breast screening program. Med Phys.

[17] Abdullah AK, Kelly J, Thompson JD, Mercer CE, Aspin R, Hogg P (2017) The impact of simulated motion blur on lesion detection performance in full-field digital mammography. Br J Radiol 90:20160871 10.1259/bjr.20160871 PMC5594981 28508724

[18] Saunders RS, Baker JA, Delong DM, Johnson JP, Samei E (2007) Does image quality matter? Impact of resolution and noise on mammographic task performance. Med Phys 34:3971–3981 10.1118/1.2776253 17985642

[19] Halling-Brown MD, Warren LM, Ward D et al (2020) OPTIMAM mammography image database: a large scale resource of mammography images and clinical data. arXiv:2004.04742 10.1148/ryai.2020200103 PMC8082293 33937853

[20] Mackenzie A, Dance DR, Workman A, Yip M, Wells K, Young KC (2012) Conversion of mammographic images to appear with the noise and sharpness characteristics of a different detector and x-ray system. Med Phys 39:2721–2734 10.1118/1.4704525 22559643

[21] Mackenzie A, Dance DR, Diaz O, Young KC (2014). Image simulation and a model of noise power spectra across a range of mammographic beam qualities. Med Phys.

[22] Mackenzie A, Dunn HL, Boita J (2019). A method to modify mammography images to a appear as if acquired using different radiographic factors. Proc SPIE.

[23] Håkansson M, Svensson S, Zachrisson S, Svalkvist A, Båth M, Månsson LG (2010) VIEWDEX: an efficient and easy-to-use software for observer performance studies. Radiat Prot Dosimetry 139:42–51 10.1093/rpd/ncq057 20200105

[24] Mackenzie A, Warren LM, Wallis MG (2016). Breast cancer detection rates using four different types of mammography detectors. Eur Radiol.

[25] Sund P, Båth M, Kheddache S, Månsson LG (2004). Comparison of visual grading analysis and determination of detective quantum efficiency for evaluating system performance in digital chest radiography. Eur Radiol.

[26] Séradour B, Heid P, Estève J (2014). Comparison of direct digital mammography, computed radiography, and film-screen in the French national breast cancer screening program. AJR Am J Roentgenol.

[27] Boita J, Mackenzie A, Sechopoulos I (2019) Validation of a method to simulate the acquisition of mammographic images with different techniques. Proc SPIE 10948:109481K

