# Supplementary material for: How does image quality affect radiologists’ perceived ability for image interpretation and lesion detection in digital mammography?
Source: Eur Radiol. 2021 Jan 21;31(7):5335–43. doi: 10.1007/s00330-020-07679-8 (PMC8213590; doi:10.1007/s00330-020-07679-8)
Supplement: Supplementary file 1 — (DOCX 180 kb) [file 330_2020_7679_MOESM1_ESM.docx]

**Supplementary Material to: “How does image quality affect radiologists’ perceived ability for image interpretation and lesion detection in digital mammography?”**

Image degradation procedures

***Lower Spatial Resolution***

Spatial resolution was decreased by simulating different levels of resolution that can be found in the clinical setting. Using previously developed algorithms [1], the modulation transfer function (MTF) from the reference system was modified to resemble the sharpness of six other different system types (Figure 1S). The MTF from the reference system and from the other six systems were measured during quality control tests. Each MTF corresponded to a different level of resolution, starting from the reference system (a direct detector system, denoted a-Se I), to the system with lowest resolution, a digital radiography (DR) system with an indirect detector (denoted chest CsI). The MTF of the latter system was included to achieve a very low resolution.


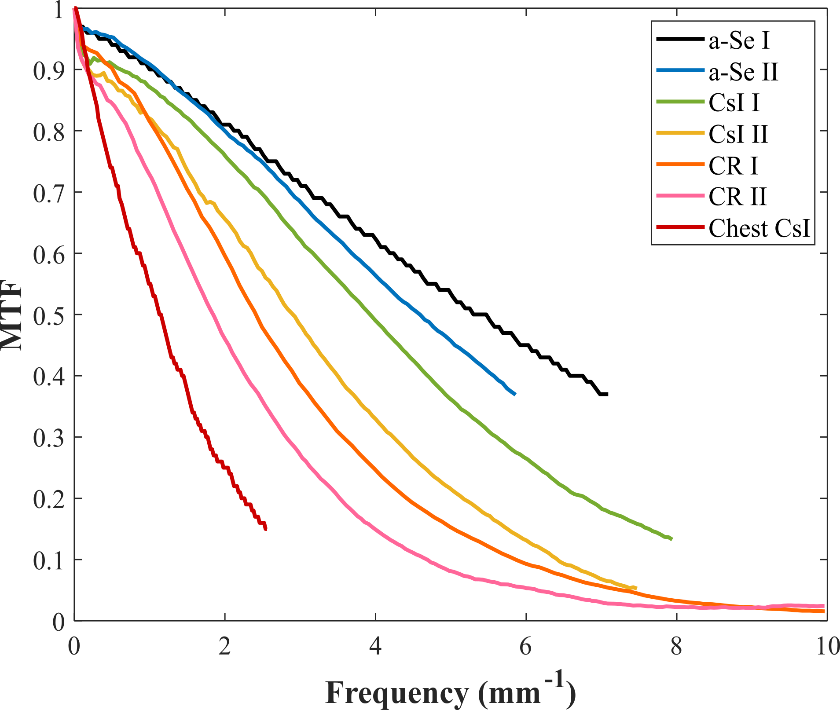


Figure 1S. Modulation transfer functions measured for seven different digital mammography systems (including direct detector (a-Se), indirect detector (CsI), and computed radiography (CR) mammography systems) and one chest x-ray indirect detector system used to simulate the six levels of lower spatial resolution.

*Higher and Lower Contrast*

The contrast was changed by modifying the presentation of each image on the monitor. A presentation function was defined as a sigmoid function that allowed for the adjustment of the contrast by changing the shape of the curve. Selecting a sigmoid function instead of a more common linear presentation function allows for a smoother change of contrast and avoids clipping. To decrease the contrast, the slope of the curve was decreased. On the other hand, to increase the contrast, the presentation curve was made steeper to intensify the local contrast in the structures. Figure 2S shows examples of sigmoid functions for decreasing and increasing of contrast.


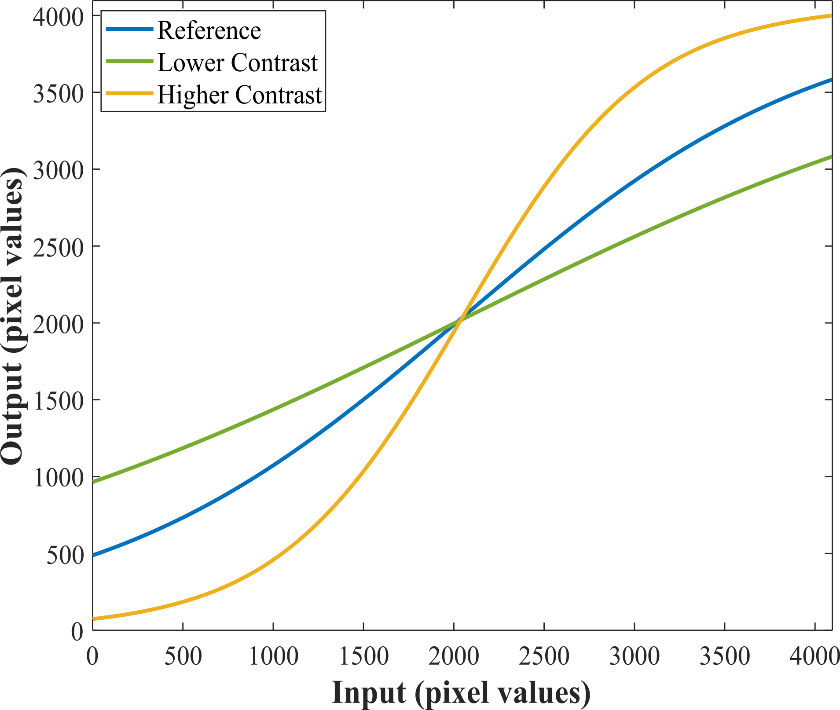


Figure 2S. Exemplification of the sigmoid functions calculated for decreasing and increasing of contrast.

*Correlated and Quantum Noise*

The texture of the image was changed by adding two different types of noise: correlated noise and quantum noise.

Correlated noise was simulated by first increasing the correlation in the pixel values in a homogeneous image of a 45 mm thick PMMA slab, acquired using a Lorad Selenia mammography system. The image was acquired using the automatic exposure control (AEC)-selected settings. To increase the noise correlation, a Butterworth high pass filter was applied to the homogeneous image to enhance the higher frequencies. The cut-off frequency of 1.8 mm^-1^ was selected to approximately match the frequency characteristics of correlated noise found in images from previously failing systems. The mean of the resultant noise image was subtracted from the image, and the resulting zero-mean noise image was added to the mammographic image being corrupted.

Quantum noise was added by simulating images as if they were acquired at different dose levels using an algorithm previously developed by Mackenzie *et al* [1–3] and validated by Boita *et al* [4]. The algorithm takes into account the differences in noise between the reference and the modified images as well as other characteristics such as scatter, tube voltage, tube current-exposure time product, anode/filter combination, etc. The reference image was modified to create new images corresponding to dose reduction levels of 15%, 30%, 45%, 60%, 75%, and 80%.

Score Variability Results

The distribution of the radiologist index across cases and for most types of degradation did not vary considerably, i.e., the values of the average of the standard deviation of the index calculated for each radiologist are lower than 1, as seen in Table 1S for calcification and soft tissue cases.

Table 1S. Average of the standard deviation of the index calculated for each radiologist across calcification and soft tissue cases and for the five types of degradation.

| **Types of Degradation** | **Calcifications** | **Soft Tissue** |
| --- | --- | --- |
| Resolution | 0.94 | 1.05 |
| Quantum Noise | 0.84 | 0.77 |
| Correlated Noise | 0.83 | 1.31 |
| High Contrast | 0.42 | 0.80 |
| Low Contrast | 0.27 | 0.81 |

REFERENCES

[1] Mackenzie A, Dance DR, Workman A, Yip M, Wells K, Young KC. Conversion of mammographic images to appear with the noise and sharpness characteristics of a different detector and x-ray system. Med Phys 2012;39:2721–34.

[2] Mackenzie A, Dance DR, Diaz O, Young KC. Image Simulation and a Model of Noise Power Spectra Across a Range of Mammographic Beam Qualities. Med Phys 2014;41.

[3] Mackenzie A, Dunn HL, Boita J, Dance DR, Young KC. A method to modify mammography images to a appear as if acquired using different radiographic factors. Proc. SPIE, vol. 10948, SPIE; 2019, p. 109482F.

[4] Boita J, Mackenzie A, Sechopoulos I. Validation of a method to simulate the acquisition of mammographic images with different techniques. Proc. SPIE, vol. 10948, SPIE; 2019, p. 109481K.
